# Sociodemographic Factors Associated with Purchasing Frozen, Fresh, Canned, and Dried Produce in a Nationally Representative Sample of United States Households

**DOI:** 10.1016/j.cdnut.2024.104528

**Published:** 2024-12-15

**Authors:** Graham E Bastian, Joslyn K Russell, Annie J Roe, Raveen Rani

**Affiliations:** 1 School of Health and Human Sciences, South Dakota State University, Brookings, SD, United States; 2 Department of Biology and Microbiology, South Dakota State University, Brookings, SD, United States; 3 Margaret Ritchie School of Family and Consumer Sciences, University of Idaho, Moscow, ID, United States

**Keywords:** frozen foods, sociodemographic factors, food assistance, secondary data analysis, consumer behavior

## Abstract

**Background:**

Frozen fruits and vegetables (FV) are convenient, cost-effective, reduce food waste, and can be just as nutritious as their fresh counterparts. Despite these numerous advantages, it is unclear which consumer segments are more likely to purchase frozen FV, especially compared with fresh, canned, or dried FV, which could help inform targeted nutrition education interventions.

**Objective:**

The objective of this study is to explore sociodemographic factors associated with increased or decreased odds of purchasing frozen, fresh, canned, and dried FV in a nationally representative sample.

**Methods:**

A secondary data analysis was conducted using publicly available data from the nationally representative 2021 United States Bureau of Labor Statistics Consumer Expenditure Diary Surveys, in which participants were instructed to record all household expenditures during a 2-wk timeframe. Chi-square analyses and unadjusted and adjusted logistic regressions were used to explore the associations between the included sociodemographic variables and FV purchasing.

**Results:**

Of the final sample (*n* = 6028), 230 purchased frozen fruits and 1163 purchased frozen vegetables during the study period. Households with higher income, higher educational attainment, and more children <18 y had higher odds of purchasing any type of FV. Households utilizing the Supplemental Nutrition Assistance Program (SNAP) had higher odds of purchasing frozen vegetables after controlling for other variables (odds ratio: 1.24, 95% confidence interval: 0.99, 1.56, *P* = 0.07). Compared with White non-Hispanic-led households, Asian- and Hispanic-led households had higher odds of purchasing fresh FV and lower odds of purchasing frozen vegetables.

**Conclusions:**

The results of this exploratory study could inform future research, particularly regarding the factors that influence the frozen FV perceptions of SNAP consumers and Hispanic and Asian households. Since nutrition educators who teach SNAP participants already promote frozen FV, studies that investigate how SNAP consumers’ perceptions change because of such interventions are also warranted.

## Introduction

Despite the numerous health benefits associated with eating fruits and vegetables (FV) [1], roughly 90% of United States adults fail to meet FV recommendations from the *Dietary Guidelines for Americans* [2,3]. Further disparities regarding FV intake exist among certain sociodemographic groups, including men, young adults, and adults with low incomes [2]. FV are typically purchased in fresh, frozen, and canned varieties; fruits are also purchased in dried forms (for example, raisins). Most research on FV purchasing and consumption measures all forms of FV in aggregate, making sociodemographic comparisons among FV varieties difficult. Notably, consumers with low incomes utilizing the Supplemental Nutrition Assistance Program (SNAP) have been shown to be more likely to purchase frozen, canned, and dried FV within 1 wk of receiving benefits and less likely to purchase fresh FV within 1 wk of benefits renewal [4].

Among the FV varieties, frozen FV may be uniquely positioned to be a convenient option to help consumers, especially those with low incomes, improve FV intake and diet quality. They have been shown to have similar nutrition profiles to their fresh counterparts [5, 6]. Moreover, consumers who ate more frozen FV were shown to have higher overall FV intakes compared with their peers, as well as higher potassium and lower sodium intakes [7]. Frozen FV are also less likely to generate food waste [8], and although frozen FV may not always be less expensive than their fresh counterparts [6], resulting in less waste may make frozen FV a more cost-effective option to consumers with low income, like SNAP participants. All the aforementioned points are used by nutrition educators, including those prioritizing audiences with low income in programs like the Supplemental Nutrition Assistance Program—Education (SNAP-Ed) and the Expanded Food and Nutrition Education Program (EFNEP), to educate consumers that frozen FV, especially those lower in added sugars, solid fats, and sodium, are nutritious and budget-friendly choices [for example, [[9], [10], [11]]]. In fact, a common refrain among nutrition educators is that when it comes to FV, “All Forms Fit,” including fresh, frozen, canned, and dried [for example, [[12], [13], [14]]].

Despite this promotion of frozen FV to audiences with low incomes, it is unclear whether consumers with limited budgets and/or using SNAP are more likely to purchase these foods. This is of particular concern because policy decisions have been proposed or made to affect the foods available to SNAP consumers without an understanding of how it would impact their shopping choices. For instance, the United States Department of Agriculture (USDA)’s Gus Schumacher Nutrition Incentive Program allows recipients of its Nutrition Incentive Program grants to offer incentives of fresh, frozen, or canned FV to SNAP consumers [15]; however, the Produce Prescription Program grantees are only allowed to provide fresh FV to their participants with low income [15], even if offering frozen or canned would allow the produce to last longer or reduce food waste. At the time of writing, the Supporting All Healthy Options When Purchasing Produce Act has been proposed to expand the types of produce offered by this program [16]. Yet, as the Farm Bill’s largest program [17], SNAP is often the target of bills at the state and federal levels that aim to limit eligibility requirements, benefit amounts, and consumer choice.

The purpose of this exploratory study was to identify sociodemographic factors, including household income and SNAP participation, that are associated with the purchase of frozen, fresh, canned, and dried FV. By better understanding current consumer trends in FV purchases, policies can be proposed that better fit the needs of consumers, including those on SNAP and other food assistance programs. Moreover, it would help nutrition educators identify potential audiences that could benefit from targeted education on the nutritional benefits of certain types of FV, like frozen. The results of this exploratory analysis could also be used by other researchers for future hypothesis generation and testing.

## Methods

### Study design and participants

This study was a secondary analysis of data from the United States Bureau of Labor Statistics Consumer Expenditure Surveys (CES) [18]. These are 2 surveys administered quarterly to assess the demographic characteristics, income, and expenditures of United States consumers. The CES are divided into 2 separate surveys. The Interview Survey is used to track large and/or recurring consumer expenditures (for example, automobiles and rent). Participants are asked to report all such purchases made in the past 3 mo. The Diary Survey is used to track smaller, more frequently purchased items that are more difficult for consumers to recall (for example, food and gasoline). Participants report all such purchases in a 2-wk, prospective diary format. Both surveys randomly sample United States households and their data are representative of the United States civilian, noninstitutionalized population (roughly 98% of the national population). Together, the 2 surveys are used to inform economic research and policy by several United States federal departments, including the USDA, Internal Revenue Service, and Department of Defense [19].

Although data from the CES are disseminated in aggregated reports, Public Use Microdata are also provided online, which include individual response data, excluding personally identifiable information [20]. These data are free to use for researchers. As this study aimed to explore grocery purchases related to FV, Public Use Microdata were used specifically from the CES Diary Survey. As the CES’s methodology ensures that the surveyed sample is nationally representative, sample weights are not needed when analyzing the Public Use Microdata at the national level. All households who completed the Diary Survey in 2021 were included in the study, as long as the age of the reference person completing the survey on behalf of the household was ≥18 y. This study was reviewed by South Dakota State University’s Institutional Review Board and determined to be exempt from full board review (approval #IRB-2305001-EXM).

### Measures

#### Purchase diaries

The Diary Survey data are 2-wk, prospective records of consumer expenditures. Once data collection is completed, purchases reported by the participants are then coded by the United States Bureau of Labor Statistics into previously identified universal classification codes (UCCs), which are outlined in Table 1. These codes were used to create 7 discrete categories: frozen fruits, fresh fruits, canned fruits, dried fruits, frozen vegetables, fresh vegetables, and canned vegetables. All expenditures were reported in United States dollars (USD).

**TABLE 1:** Fruit and vegetable universal classification codes (UCCs) used from the consumer expenditure surveys for secondary analysis and accompanying descriptive statistics.

UCC	Description	Category for secondary analysis	Number (%) of households (*n* = 6028) that made ≥1 purchase in category	Mean 2-wk total from purchasing subsample (SD)	Minimum and maximum purchases
**130121**	Frozen fruit	Frozen fruits	230 (3.81)	$9.30 (9.41)	$1.09, $103.91
**110110**	Apples	Fresh fruits	2411 (40.00)	$8.81 (7.69)	$0.33, $135.77
**110210**	Bananas				
**110310**	Oranges				
**110410**	Other fresh fruits				
**110510**	Citrus fruits, excluding oranges				
**130310**	Canned fruits	Canned fruits	427 (7.08)	$4.56 (3.30)	$0.24, $27.18
**130320**	Dried fruit	Dried fruits	150 (2.49)	$5.74 (4.13)	$0.37, $27.92
**140110**	Frozen vegetables	Frozen vegetables	1163 (19.29)	$6.52 (5.90)	$0.40, $88.14
**120110**	Potatoes	Fresh vegetables	2410 (39.98)	$6.95 (6.28)	$0.18, $74.00
**120210**	Lettuce				
**120310**	Tomatoes				
**120410**	Other fresh vegetables				
**140210**	Canned beans	Canned vegetables	1044 (17.32)	$6.13 (5.27)	$0.07, $58.58
**140220**	Canned corn				
**140230**	Canned miscellaneous vegetables				

#### Sociodemographic factors

Diary Survey participants reported on several sociodemographic factors that were used for this study. Some demographics from the reference person who completed the survey on the household’s behalf (assumed to be the head of the household) were used, including educational attainment, race (self-reported from White, Black, Native American, Asian, Pacific Islander, and Multi-race), and ethnicity (Hispanic or non-Hispanic). Race and ethnicity categories were reported as is, without combining groups, per the *Journal of the American Medical Association’s* race and ethnicity reporting guidelines, even when categorical cells for some of the racial groups were too small to be used in regression analyses [21]. Other factors that described the household were used, including total annual household income, in USD, before taxes (UCC 980000), the annual allotment of SNAP benefits, in USD (UCC 900150), and the number of children in the household < 18 y (UCC 980050).

### Statistical analysis

All statistical analyses were conducted using SAS 9.4 [22]. The data used for the analyses were compiled from 12 separate data sets from the Public Use Microdata with the following file names: FMLD (which contained most of the demographic data), DMDB (which included income and SNAP data, as well as the number of children in the household), and EXPD (which included expenditure data, specifically for the FV), for each of the 4 quarters. The data were then cleaned to remove households with a reference person who was <18 y and households that reported no income in the past 12 mo, resulting in a final sample of 6028.

Households who reported receiving any SNAP benefits over the past 12 mo were considered SNAP-participating households for the analyses. Educational attainment was recoded as 1 = less than high school, 2 = high school graduate, 3 = some college or associate degree, 4 = Bachelor’s degree, and 5 = master’s degree or higher. All expenditures of FV over the 2-wk diary period were summed for each household by type (frozen, fresh, canned, and dried). Because the Diary Survey data only collect purchase data for 2 wk, the distributions for the amount purchased on each type of produce were all heavily right-skewed (that is, a considerable proportion of the sample did not report buying these foods in their respective 2-wk window, and therefore, the mean, median, and mode of each distribution were 0). Thus, for the purpose of this study, households underwent binary categorization where those who had FV expenditures >$0 for any type were considered “purchasers” of that food.

Descriptive analyses were conducted on the whole sample, and χ^2^ analyses were conducted to determine whether households that purchased each specific FV type had sociodemographic data that differed from the full sample. Then, logistic regressions were conducted to determine the odds of purchasing each type of FV for each sociodemographic characteristic. Both unadjusted models and models adjusted for all other variables in the study were conducted, to explore the relationships that the sociodemographic variables had with each other. No multicollinearity was present in any of the models.

## Results

A total of 6028 households were included in the sample. The mean age of the reference person completing the Diary Survey for the household was 53.1 y; the mean household income was $90,258 with a median of $62,327. The average number of purchasing households and the average purchasing amounts for each type of FV are detailed in Table 1. Although only <4% of households purchased frozen fruits during the study period, this FV category had the highest mean purchase of $9.30. Roughly 1 in 5 households purchased frozen vegetables during the study period, whereas 2 in 5 households purchased fresh fruits or fresh vegetables.

The sample’s descriptive characteristics are further detailed in Table 2. Approximately two-thirds of the household heads were White non-Hispanic; 71% had at least some postsecondary education; 10% received SNAP benefits within the last 12 mo; and 26% had ≥1 child <18 y in the household. The subsamples of households that purchased FV had greater proportions of higher-income households, households with children, and higher educational attainment of household heads (Table 2). Regarding race and ethnicity, the subsample of frozen fruit purchasers had a higher proportion of Asian household heads and a lower proportion of Hispanic household heads. Both Asian- and Hispanic-led households had higher representation among fresh fruit and fresh vegetable purchasers (Table 2).

**TABLE 2:** Descriptive characteristics of the 2021 consumer expenditure survey 2-wk diary participants.

	Full sample	Frozen fruit purchasers	Fresh fruit purchasers	Canned fruit purchasers	Dried fruit purchasers	Frozen vegetable purchasers	Fresh vegetable purchasers	Canned vegetable purchasers
**Age (y) mean (SD)**	53.14 (17.41)	51.69 (16.48)	53.55 (16.83)	54.89 (15.99)	55.35 (17.74)	52.36 (16.61)	53.19 (16.70)	52.68 (15.72)
*P* value^1^	–	0.21	0.32	0.04	0.12	0.16	0.90	0.42
**Annual income, mean (SD)**	$90,258.20 ($89,428.84)	$123,709.97 ($112,287.17)	$101,525.77 ($97,263.84)	$111,782.85 ($106,470.87)	$105,896.09 ($106,611.24)	$105,657.02 ($98,182.83)	$100,636.20 ($95,725.51)	$107,100.45 ($97,658.70)
*P* value^1^	–	0.00	0.00	0.00	0.04	0.00	0.00	0.00
**Education (%)**^2^								
Less than high school	8.16	5.22	7.30	5.85	4.67	6.28	7.47	6.80
High school graduate	20.52	15.22	17.54	17.56	14.67	19.09	18.38	19.25
Some college or associate degree	28.48	23.48	27.17	26.23	22.00	27.17	26.47	27.30
Bachelor’s degree	26.18	26.52	27.87	30.91	33.33	27.34	27.97	25.29
Master’s degree or higher	16.66	29.57	20.12	19.44	25.33	20.12	19.71	21.36
*P* value^1^	–	0.00	0.00	0.02	0.00	0.00	0.00	0.00
**Race and ethnicity (%)**^2^								
White, non-Hispanic	66.71	68.26	65.86	69.32	68.67	69.65	66.06	69.44
Black, non-Hispanic	11.13	10.43	9.58	10.77	10.00	11.01	9.46	8.72
Native American, non-Hispanic	0.46	0.43	0.46	0.23	0	0.43	0.41	0.38
Asian, non-Hispanic	6.52	11.74	7.63	6.79	10.00	5.59	7.88	6.03
Pacific Islander, non-Hispanic	0.33	0	0.33	0.23	0	0.43	0.29	0.38
Multiracial, non-Hispanic	1.29	0.43	1.24	1.17	1.33	1.72	1.41	1.25
Hispanic, any race	13.55	8.70	14.89	11.48	10.00	11.18	14.48	13.79
*P* value^1^	–	0.01	0.00	0.84	0.45	0.06	0.00	0.18
**SNAP participants (%)**^3^	9.59	5.65	8.88	6.56	6.67	10.15	8.88	7.76
*P* value^1^	–	0.39	0.13	0.03	0.22	0.47	0.13	0.03
**Number of children in the household, mean (SD)**	0.48 (0.95)	0.67 (1.00)	0.55 (1.01)	0.59 (1.02)	0.61 (1.14)	0.62 (1.07)	0.55 (1.01)	0.59 (1.01)
*P* value^1^	–	0.00	0.00	0.02	0.10	0.00	0.00	0.00
**Total observations**	6028	230	2411	427	150	1163	2410	1044

1 Results of either a *t*-test (continuous variables) or a χ^2^ test (categorical variables) compared with the full sample.

2 Reported by the main reference household member who completed the survey.

3 SNAP = Supplemental Nutrition Assistance Program. Households were considered SNAP participants if any SNAP benefit income was reported from the past 12 mo.

The unadjusted and adjusted models for fruit and vegetable purchasing are displayed in TABLE 3, TABLE 4, respectively. Income was positively associated with purchasing all types of FV, but the magnitude of these associations was low, given the size of the regression coefficients. Associations with household head ages, when present, were very minor. The household head’s educational attainment and the number of children in the household were both positively associated with purchasing each FV type. Educational attainment seemed to be somewhat attenuated in the adjusted models, after controlling for other variables like income, particularly for canned fruits and frozen vegetables (TABLE 3, TABLE 4). The number of children in the household was positively associated with purchasing FV of all types; for each additional child in the household, the odds of purchasing fruits increased by 15%–25%, depending on type, and the odds of purchasing vegetables increased by 12%–19% (TABLE 3, TABLE 4).

**TABLE 3:** Factors associated with purchasing frozen, fresh, canned, and dried fruits among participants of the 2021 consumer expenditure survey 2-wk purchase diary (*n* = 6028).

	Frozen fruits		Fresh fruits		Canned fruits		Dried fruits	
	Unadjusted *β*_1_	Adjusted *β*_1_^1^	Unadjusted β_1_	Adjusted *β*_1_^1^	Unadjusted *β*_1_	Adjusted *β*_1_^1^	Unadjusted *β*_1_	Adjusted *β*_1_^1^
Annual income^2^^,^^3^	3.17E–6	1.97E–6	2.32E–6	1.76E–6	2.35E–6	2.02E–6	1.68E–6	5.01E–7

	Unadjusted OR	Adjusted OR^1^	Unadjusted OR	Adjusted OR^1^	Unadjusted OR	Adjusted OR^1^	Unadjusted OR	Adjusted OR^1^

**Age**^2^	1.00 (0.99, 1.00)	1.00 (0.99, 1.01)	1.00 (1.00, 1.01)	1.01 (1.01, 1.01)	1.01 (1.00, 1.01)	1.01 (1.01, 1.02)	1.01 (1.00, 1.02)	1.02 (1.00, 1.03)
**Education**								
Less than high school	0.34 (0.18, 0.64)	0.52 (0.27, 1.02)	0.60 (0.48, 0.74)	0.61 (0.48, 0.78)	0.59 (0.38, 0.94)	0.74 (0.45, 1.22)	0.37 (0.16, 0.83)	0.40 (0.17, 0.94)
High school graduate	0.40 (0.26, 0.61)	0.56 (0.35, 0.86)	0.56 (0.47, 0.66)	0.63 (0.52, 0.75)	0.72 (0.52, 0.99)	0.89 (0.63, 1.26)	0.46 (0.27, 0.78)	0.51 (0.29, 0.89)
Some college or associate degree	0.45 (0.31, 0.65)	0.58 (0.39, 0.85)	0.66 (0.56, 0.77)	0.75 (0.63, 0.88)	0.77 (0.58, 1.04)	0.95 (0.70, 1.29)	0.50 (0.31, 0.80)	0.55 (0.34, 0.91)
Bachelor’s degree	0.55 (0.39, 0.79)	0.62 (0.43, 0.89)	0.79 (0.68, 0.93)	0.85 (0.73, 1.00)	1.01 (0.76, 1.35)	1.14 (0.85, 1.52)	0.83 (0.54, 1.28)	0.89 (0.58, 1.38)
Master's degree or higher	REF	REF	REF	REF	REF	REF	REF	REF
**Race and ethnicity**^2^^,^^4^								
White, non-Hispanic	REF	REF	REF	REF	REF	REF	REF	REF
Black, non-Hispanic	0.91 (0.59, 1.41)	1.03 (0.66, 1.60)	0.80 (0.68, 0.96)	0.89 (0.75, 1.06)	0.93 (0.67, 1.28)	1.07 (0.77, 1.48)	0.87 (0.50, 1.51)	0.98 (0.56, 1.71)
Native American, non-Hispanic	–	–	0.99 (0.46, 2.12)	0.98 (0.45, 2.14)	–	–	–	–
Asian, non-Hispanic	1.82 (1.19, 2.77)	1.53 (1.00, 2.36)	1.35 (1.10, 1.66)	1.27 (1.03, 1.57)	1.00 (0.68, 1.49)	0.97 (0.65, 1.45)	1.51 (0.87, 2.62)	1.39 (0.79, 2.44)
Pacific Islander, non-Hispanic	–	–	1.02 (0.42, 2.51)	1.05 (0.43, 2.61)	–	–	–	–
Multi-racial, non-Hispanic	–	–	0.96 (0.60, 1.52)	0.96 (0.60, 1.54)	0.86 (0.35, 2.15)	0.91 (0.36, 2.28)	–	–
Hispanic, any race	0.62 (0.39, 0.99)	0.71 (0.43, 1.16)	1.20 (1.03, 1.40)	1.43 (1.21, 1.68)	0.80 (0.59, 1.10)	0.99 (0.71, 1.37)	0.71 (0.41, 1.23)	0.93 (0.52, 1.65)
**SNAP participation**^5^	1.80 (1.02, 3.18)	0.72 (0.40, 1.30)	1.15 (0.96, 1.37)	1.04 (0.86, 1.68)	1.55 (1.05, 2.30)	0.77 (0.51, 1.16)	1.50 (0.78, 2.86)	0.87 (0.44, 1.72)
**Number of children in household**	1.19 (1.06, 1.34)	1.18 (1.04, 1.35)	1.13 (1.07, 1.19)	1.15 (1.08, 1.22)	1.13 (1.03, 1.24)	1.21 (1.09, 1.34)	1.14 (0.98, 1.32)	1.25 (1.06, 1.48)

Model fit estimate								

**Akaike information criterion (AIC)**	–	1925.29	–	7999.18	–	3064.80	–	1403.49

Abbreviations: OR, odds ratio; REF, reference category.

1 Adjusted models included all listed variables.

2 Reported by the main reference household member who completed the survey.

3 All *P* < 0.0001.

4 Some racial categories had insufficient cell sizes to be included in some of the models; thus, those results were not presented.

5 SNAP = Supplemental Nutrition Assistance Program. Households were considered SNAP participants if any SNAP benefit income was reported from the past 12 mo. The reference category was no SNAP participation.

**TABLE 4:** Factors associated with purchasing frozen, fresh, and canned vegetables among participants of the 2021 consumer expenditure survey 2-wk purchase diary (*n* = 6028).

	Frozen vegetables		Fresh vegetables		Canned vegetables	
	Unadjusted *β*_1_	Adjusted *β*_1_^1^	Unadjusted *β*_1_	Adjusted *β*_1_^1^	Unadjusted *β*_1_	Adjusted *β*_1_^1^
Annual income^2^^,^^3^	2.13E–6	1.70E–6	2.14E–6	1.57E–6	2.24E–6	1.77E–6

	Unadjusted OR	Adjusted OR^1^	Unadjusted OR	Adjusted OR^1^	Unadjusted OR	Adjusted OR^1^

**Age**^2^	1.00 (0.99, 1.00)	1.00 (1.00, 1.01)	1.00 (1.00, 1.00)	1.01 (1.00, 1.01)	1.00 (0.99, 1.00)	1.00 (1.00, 1.01)
**Education**						
Less than high school	0.57 (0.43, 0.77)	0.67 (0.49, 0.91)	0.64 (0.52, 0.80)	0.69 (0.54, 0.87)	0.59 (0.44, 0.79)	0.69 (0.50, 0.95)
High school graduate	0.72 (0.59, 0.89)	0.83 (0.67, 1.04)	0.62 (0.52, 0.74)	0.71 (0.60, 0.85)	0.68 (0.55, 0.84)	0.81 (0.64, 1.01)
Some college or associate degree	0.74 (0.61, 0.90)	0.83 (0.68, 1.02)	0.66 (0.56, 0.77)	0.75 (0.63, 0.88)	0.70 (0.57, 0.85)	0.80 (0.65, 0.99)
Bachelor’s degree	0.83 (0.69, 1.01)	0.89 (0.73, 1.08)	0.83 (0.71, 0.97)	0.89 (0.76, 1.04)	0.70 (0.58, 0.86)	0.75 (0.61, 0.92)
Master’s degree or higher	REF	REF	REF	REF	REF	REF
**Race and ethnicity**^2^^,^^4^						
White, non-Hispanic	REF	REF	REF	REF	REF	REF
Black, non-Hispanic	0.93 (0.76, 1.15)	0.97 (0.78, 1.20)	0.79 (0.66, 0.93)	0.85 (0.71, 1.01)	0.71 (0.56, 0.90)	0.77 (0.61, 0.98)
Native American, non-Hispanic	0.86 (0.33, 2.27)	0.77 (0.29, 2.07)	0.85 (0.39, 1.84)	0.85 (0.39, 1.86)	-	-
Asian, non-Hispanic	0.79 (0.60, 1.04)	0.71 (0.54, 0.94)	1.43 (1.16, 1.76)	1.34 (1.09, 1.66)	0.87 (0.66, 1.15)	0.80 (0.60, 1.06)
Pacific Islander, non-Hispanic	1.32 (0.48, 3.65)	1.29 (0.46, 3.60)	0.82 (0.33, 2.06)	0.84 (0.33, 2.12)	-	-
Multi-racial, non-Hispanic	1.37 (0.82, 2.29)	1.33 (0.79, 2.24)	1.18 (0.75, 1.85)	1.19 (0.75, 1.87)	0.91 (0.50, 1.66)	0.89 (0.49, 1.63)
Hispanic, any race	0.75 (0.61, 0.92)	0.78 (0.63, 0.96)	1.14 (0.98, 1.33)	1.28 (1.09, 1.51)	0.97 (0.80, 1.19)	1.06 (0.85, 1.30)
**SNAP participation**^5^	0.93 (0.75, 1.15)	1.24 (0.99, 1.56)	1.15 (0.96, 1.37)	1.02 (0.85, 1.23)	1.32 (1.03, 1.68)	0.86 (0.67, 1.12)
**Number of children in household**	1.19 (1.12, 1.27)	1.19 (1.11, 1.27)	1.12 (1.07, 1.19)	1.12 (1.06, 1.19)	1.14 (1.07, 1.21)	1.14 (1.06, 1.22)

Model fit estimate						

**Akaike information criterion (AIC)**	–	5856.54	–	8030.54	–	5516.89

Abbreviations: OR, odds ratio; REF, reference category.

1 Adjusted models included all listed variables.

2 Reported by the main reference household member who completed the survey.

3 All *P* < 0.0001.

4 Some racial categories had insufficient cell sizes to be included in some of the models; thus, those results were not presented.

5 SNAP = Supplemental Nutrition Assistance Program. Households were considered SNAP participants if any SNAP benefit income was reported from the past 12 mo. The reference category was no SNAP participation.

Households that participated in SNAP had 24% higher odds of purchasing frozen vegetables in the adjusted model (odds ratio [OR]: 1.24, 95% confidence interval [CI]: 0.99, 1.56, *P* = 0.07). After controlling for the other variables, when compared with households led by a White non-Hispanic adult, Asian-led households had higher odds of purchasing fresh fruits (OR: 1.27, 95% CI: 1.03, 1.57, *P* = 0.03) and fresh vegetables (OR: 1.34, 95% CI: 1.09, 1.66, *P* = 0.007), and lower odds of purchasing frozen vegetables (OR: 0.71, 95% CI: 0.54, 0.94, *P* = 0.02); Hispanic-led households also had higher odds of purchasing fresh fruits (OR: 1.43, 95% CI: 1.21, 1.68, *P* < 0.001) and fresh vegetables (OR: 1.28, 95% CI: 1.09, 1.51, *P* = 0.003), and lower odds of purchasing frozen vegetables (OR: 0.78, 95% CI: 0.63, 0.96, *P* = 0.02); and Black-led households had lower odds of purchasing canned vegetables (OR: 0.77, 95% CI: 0.61, 0.98, *P* = 0.03) (TABLE 3, TABLE 4).

## Discussion

**FIGURE 1:**
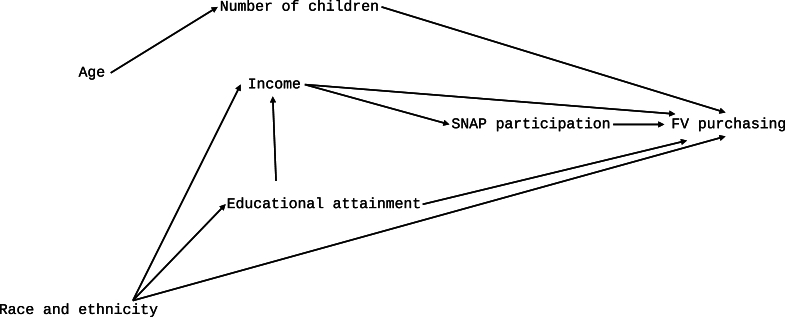
A hypothesized directed acyclic graph approximating the study variables' causal relationships. FV, fruits and vegetables; SNAP, Supplemental Nutrition Assistance Program.

Our exploratory study aimed to identify sociodemographic factors among a nationally representative sample of United States households that were associated with the purchase of FV of varying types, particularly frozen FV. The results indicated that several associations were consistent among all FV types, such as income, educational attainment, and the number of children in the household. Associations that differed among FV type (frozen, fresh, canned, and dried) were SNAP participation, which after controlling for other variables, seemed to have an association of relevant magnitude with frozen vegetables only; and race and ethnicity, which showed that FV purchasing trends, particularly among Asian and Hispanic households, differed from White non-Hispanic households. Although these studies’ results cannot imply causation outright, our findings can inform hypothesized causative pathways among the study variables to inform future hypothesis testing. A hypothesized directed acyclic graph [23] is presented in Figure 1 based on our results. We assume that the relationship between SNAP participation and FV purchasing is confounded by income; the relationship between educational attainment and FV purchasing is mediated by income; and the relationship between race and ethnicity and FV purchasing is mediated by both educational attainment and income. The number of children in a household is a competing exposure to FV purchasing with income, SNAP participation, educational attainment, and race and ethnicity; and age is an instrument to the number of children only. Future studies could test the validity of these hypothesized causal pathways.

Although this study, to the authors’ knowledge, is unique in exploring the sociodemographic factors associated with United States consumers’ purchasing of frozen FV compared with fresh, canned, and dried FV, it shares similar results with market research published in 2022 by the American Frozen Food Institute in partnership with the market research firm 210 Analytics [24]. In that report, core frozen FV buyers, defined as those purchasing daily or every few days, were more likely to be older Millennials (defined as being ages 32–41 at the time of the study), have larger households with more children, and have higher incomes. Further, the lowest-income households, which were defined as SNAP eligible, were more likely to be light consumers of frozen FV; however, the households’ actual SNAP participation was not ascertained [24]. The findings from our study help further contextualize this prior market research. In our study, higher-income households were more likely to purchase all FV types in the unadjusted and adjusted models. After controlling for income and other factors, SNAP participants had higher odds of purchasing frozen vegetables, but no other notable differences were present based on SNAP participation. Future research could further explore the characteristics of SNAP participants and income-eligible non-participants to see what factors influence FV purchases among these groups.

This study was a secondary analysis of CES data from 2021; thus, it is plausible that disruptions in daily life because of the COVID-19 pandemic influenced FV purchasing behaviors of the participating households. Some studies conducted in 2020 reported that United States consumers purchased more frozen foods during the COVID-19 pandemic [25,26], with 1 study reporting that 12% of its sample purchased a freezer during the pandemic [25]. Consumers who did purchase more frozen food during the pandemic were more likely to shop and purchase more in general [26], and considered themselves more at risk for complications from COVID-19 [25]. Notably, consumer frozen food purchase trends may have reverted to pre-pandemic levels as the novelty of the pandemic waned. Rogers et al. reported that among a longitudinal sample of 636 consumers, there was a 26% reduction in frozen food consumption between March/April 2020 and November 2020 [27]. Therefore, we speculate that this study, while using data collected during the pandemic, represented typical consumer frozen food purchasing behavior because of the data being from 2021.

SNAP was similarly affected by numerous policy changes during the pandemic to help offset increased rates of food insecurity caused by increases in unemployment [28]. Notably, emergency allotments to SNAP were authorized by USDA starting in 2020, to increase the amount of benefits that households received. By the end of 2021, 80% of states, plus the District of Columbia, Guam, and the United States Virgin Islands were still receiving SNAP emergency allotments [29]. Eligibility requirements were also adjusted in many states, allowing households with higher incomes to apply for SNAP benefits [29]. Further, in 2021 specifically, the USDA awarded an additional $69 million to fund nutrition incentive and produce prescription programs for SNAP consumers [30], which was over twice the average annual investment in such programs at the time [31]. This was part of a “COVID Relief and Response” program designed to alleviate the financial stressors that were impacting consumers during the COVID-19 pandemic. Although these projects did not start until the second half of 2021, over 54,000 customers were estimated to have been impacted by this additional funding in 2021 alone [32]. Unfortunately, because programs like nutrition incentives and produce prescriptions are not quantified in the CES, it is impossible to estimate which households, if any, in our sample were affected by these programs. Moreover, nutrition incentive and produce prescription programs have considerable variance in how they are implemented by grantees, and only some allow households to purchase frozen FV with these benefits. With these changes to the SNAP program during the time the data for our study were collected, it is difficult to ascertain whether our results regarding SNAP participation represent the program as it generally is administered, or are unique to this specific moment in time. Tseng et al. (2020), who analyzed data from 2012 to 2013, concluded in their adjusted analyses that SNAP participants were not any less likely to purchase FV than nonparticipants; however, the types that were bought depended on what part of the monthly SNAP benefit cycle the participants were in [4]. Future research could analyze CES data collected before COVID-19, or, for a more contemporary analysis, the 2024 CES data could be analyzed once available to the public, as 2024 will be the first full calendar year since COVID-19 that SNAP emergency allotments and additional nutrition incentive and produce prescription funds will not be available in any state.

A notable finding from our study was that after controlling for other factors, when compared with White non-Hispanic households, Asian- and Hispanic-led households were more likely to purchase fresh FV and less likely to purchase frozen vegetables. Several studies have noted that Hispanic/Latin American consumers view fresh produce much more favorably than frozen or canned, with freshness indicating a food’s quality and healthfulness [[33], [34], [35]]. Although research on Asian American perceptions of frozen food has been more scant, 1 study by Harrison et al. noted that low-income Chinese, Hmong, and Vietnamese Americans living in California found frozen foods to be indicative of an unhealthy, American diet and preferred fresh foods [36]. Further research could explore the cultural beliefs surrounding frozen foods, particularly frozen vegetables, in these racial and ethnic populations in greater detail, to inform ways for nutrition educators to better promote the consumption of healthy frozen foods.

This study had several strengths. First, it utilized data from the United States Bureau of Labor Statistics CES, which is nationally representative, indicating that this study has robust external validity. Second, the data were prospectively collected by participants and thus recorded exact purchase amounts; therefore, the data were not affected by recall biases common in other consumer surveys. Third, the data are publicly available, which reduced the burden of data collection for the researchers while also facilitating the study’s reproducibility. This study, however, is not without limitations. The data were cross-sectional; therefore, causation cannot be inferred from any of the associations reported in this manuscript. Furthermore, because this was a secondary data analysis, our analysis was limited by the sociodemographic variables reported in the original data set. Another limitation is that only 2 wk of purchase data for each household were used for the study, because of the design of the CES Diary Survey, so it is likely that households that do purchase a considerable amount of frozen, fresh, canned, or dried produce were miscategorized as not purchasing these foods. In particular, frozen, canned, and dried FV have long shelf lives, which may mean that households purchase these foods much less frequently than their fresh counterparts. In a similar vein, another limitation is that we had no way to determine the capacity to adequately store the different types of FV assessed in our study. Many fresh FV need to be refrigerated for proper storage, and a freezer is needed to store frozen FV. Market research from the American Frozen Food Institute indicated that although 99% of consumers have ≥1 freezer at home, 12% of consumers, including 15% of consumers with low incomes, only reported having a small freezer compartment in their refrigerator, limiting the ability to store a large quantity of frozen foods [37]. Moreover, even when freezer space is ample, consumers may be prioritizing foods other than frozen FV for the sake of food security. For instance, additional chest freezers may be bought by rural households to store food obtained from hunting or fishing so it lasts throughout the winter months [38]. Further research could explore how refrigeration and freezer space impact the FV purchasing trends of consumers with low incomes.

In conclusion, this secondary analysis of nationally representative consumer purchasing data revealed that households with higher income, higher educational attainment, and more children <18 y had higher odds of purchasing frozen, fresh, canned, and dried FV. After controlling for other factors, SNAP participants had higher odds of purchasing frozen vegetables and Asian- and Hispanic-led households were more likely to purchase fresh FV and less likely to purchase frozen vegetables. Future research can use the associations found from our study for hypothesis testing. For instance, it may be prudent to investigate the personal food values; like cost, convenience, or taste [39]; that may influence parents’, SNAP participants’, or Hispanic and Asian Americans’ decisions whether to purchase frozen foods. Nutrition educators may also wish to test interventions that would provide relevant information on frozen FV, such as their propensity to reduce food waste or their similar nutritional profiles to fresh FV, to promote their consumption. Because this type of information is already provided in federally funded community-based nutrition education programs like SNAP-Ed and EFNEP, it may also be worthwhile to investigate whether SNAP-eligible consumers who have participated in these types of lessons have more favorable perceptions around frozen FV, as well as other frozen foods, compared with SNAP-eligible consumers who have not taken these lessons.

## Author contributions

The authors’ responsibilities were as follows – GEB and AJR: designed research; GEB and JKR: conducted research; GEB and JKR: analyzed data; GEB: had primary responsibility for final content; and all authors: wrote the paper, read and approved the final manuscript.

## Data availability

Data described in the manuscript are freely available at https://www.bls.gov/cex/pumd.htm. The code book and analytic code are available upon request by contacting the corresponding author.

## Funding

This study was funded by a sponsored research opportunity from the Frozen Food Foundation. The sponsor had no involvement in the study design, but did review the manuscript before submission.

## Conflict of interest

GEB, JKR, and AJR report that financial support was provided by Frozen Food Foundation. RR declared no known competing financial interests or personal relationships that could have appeared to influence the work reported in this paper.

## References

[1] Slavin J.L., Lloyd B. (2012). Health benefits of fruits and vegetables. Adv. Nutr..

[2] Lee S.H., Moore L.V., Park S., Harris D.M., Blanck H.M. (2022). Adults meeting fruit and vegetable intake recommendations—United States, 2019, MMWR Morb. Mortal. Wkly Rep..

[3] U.S. Department of Agriculture, U.S. Department of Health and Human Services (2020). 2020–2025 Dietary Guidelines for Americans. https://www.dietaryguidelines.gov/sites/default/files/2020-12/Dietary_Guidelines_for_Americans_2020-2025.pdf.

[4] Tseng M., Mastrantonio C., Glanz H., Volpe R.J., Neill D.B., Nazmi A. (2020). Fruit and vegetable purchasing patterns and supplemental nutrition assistance program participation: findings from a nationally representative survey. J. Acad. Nutr. Diet..

[5] Li L., Pegg R.B., Eitenmiller R.R., Chun J.-Y., Kerrihard A.L. (2017). Selected nutrient analyses of fresh, fresh-stored, and frozen fruits and vegetables. J. Food Compos. Anal..

[6] Miller S.R., Knudson W.A. (2014). Nutrition and cost comparisons of select canned, frozen, and fresh fruits and vegetables. Am. J. Lifestyle Med..

[7] Storey M., Anderson P. (2018). Total fruit and vegetable consumption increases among consumers of frozen fruit and vegetables. Nutrition.

[8] Janssen A.M., Nijenhuis-de Vries M.A., Boer E.P.J., Kremer S. (2017). Fresh, frozen, or ambient food equivalents and their impact on food waste generation in Dutch households. Waste Manag.

[9] Jacobson M., Fisher L. (2024). Fresh may not always be best. https://extension.sdstate.edu/fresh-may-not-always-be-best.

[10] Irish L. (2022). SNAP-EdNY*:* how to liven up the winter with fruits and vegetables. New York State Office for the Aging.

[11] Rossi K. (2022). https://healthyfamilyct.cahnr.uconn.edu/2022/04/26/produce-picks-frozen-canned-fruits-and-vegetables/.

[12] Gatewood J. (2019). https://spendsmart.extension.iastate.edu/spendsmart/2019/11/25/all-forms-fit/.

[13] Gerber Medical Hub Fruits and veggies: all forms fit. https://medical.gerber.com/topics/fruits-and-veggies-all-forms-fit#:%7E:text=Complementary%20foods%20are%20vital%20for,%2C%20convenience%2C%20and%20cost%20factors.

[14] Cochran N., Nassonova M. (2020). https://fruitsandveggies.org/stories/heart-to-heart-with-fruits-and-veggies/.

[15] U.S. Department of Agriculture, Gus Schumacher Nutrition Incentive Program (GusNIP). [Internet]. [cited 26 June 2024]. Available from: https://www.nifa.usda.gov/grants/programs/hunger-food-security-programs/gus-schumacher-nutrition-incentive-program..

[16] (2024). H.R.3127 - SHOPP Act.

[17] U.S. Department of Agriculture. Farm bill. [Internet]. [cited 25 October 2024]. Available from: https://www.usda.gov/farmbill..

[18] U.S. Bureau of Labor Statistics. Consumer expenditure surveys. [Internet]. [cited 4 June 2024]. Available from: https://www.bls.gov/cex/..

[19] U.S. Bureau of Labor Statistics. Handbook of Methods, Consumer Expenditures and Income. [Internet]. [cited 4 June 2024]. Available from: https://www.bls.gov/opub/hom/cex/home.htm..

[20] U.S. Bureau of Labor Statistics. Public Use Microdata (PUMD). [Internet]. [cited 4 June 2024]. Available from: https://www.bls.gov/cex/pumd.htm..

[21] Flanagin A., Frey T., Christiansen S.L. (2021). AMA Manual of Style Committee, Updated guidance on the reporting of race and ethnicity in medical and science journals. JAMA.

[22] SAS Institute Inc (2013).

[23] Digitale J.C., Martin J.N., Glymour M.M. (2022). Tutorial on directed acyclic graphs. J. Clin. Epidemiol..

[24] American Frozen Food Institute (2022).

[25] Bender K.E., Badiger A., Roe B.E., Shu Y., Qi D. (2022). Consumer behavior during the COVID-19 pandemic: an analysis of food purchasing and management behaviors in U.S. households through the lens of food system resilience. Socioecon. Plann. Sci..

[26] Chenarides L., Grebitus C., Lusk J.L., Printezis I. (2021). Food consumption behavior during the COVID-19 pandemic. Agribusiness (N Y N Y).

[27] Rogers A.M., Lauren B.N., Woo Baidal J.A., Ozanne E.M., Hur C. (2021). Persistent effects of the COVID-19 pandemic on diet, exercise, risk for food insecurity, and quality of life: a longitudinal study among U.S. adults. Appetite.

[28] Kim-Mozeleski J.E., Pike Moore S.N., Trapl E.S., Perzynski A.T., Tsoh J.Y., Gunzler D.D. (2023). Food insecurity trajectories in the US during the first year of the COVID-19 pandemic. Prev. Chronic. Dis..

[29] Pukelis K. (2024). SNAP policies and enrollment following the COVID-19 pandemic. https://kelseypukelis.com/files/Pukelis_SNAPcovid.pdf.

[30] U.S. Department of Agriculture (2021). USDA invests $69 million to support critical food and nutrition security needs. https://www.usda.gov/media/press-releases/2021/08/17/usda-invests-69-million-support-critical-food-and-nutrition.

[31] Nutrition Incentive Hub (2020). GusNIP NTAE center and nutrition incentive hub to partner with 30 new GusNIP award recipients. https://www.nutritionincentivehub.org/news-events/news/2020-gusnip-award-recipients.

[32] NTAE GusNIP (2023). Gus Schumacher Nutrition Incentive Program (GusNIP): impact findings Y3: September 1, 2021 to August 31, 2022. https://www.nutritionincentivehub.org/media/2uwlf3ch/gusnip-y3-impact-findings-report.pdf.

[33] Quick V., Errickson L., Bastian G., Chang G., Davis S., Capece A. (2022). Preserving farm freshness: consumer preferences for local value-added products at urban farmers markets. J. Agric. Food Syst. Commun. Dev..

[34] Pare E.R., Body K., Gilstorf S., Lucarelli J. (2019). Qualitative focus groups: perceived influences on decision making about diet and physical activity among Hispanic/Latino participants, Health Promot. Pract.

[35] Crusan A., Roozen K., Godoy-Henderson C., Zamarripa K., Remache A. (2023). Using community-based participatory research methods to inform the development of medically tailored food kits for Hispanic/Latine adults with hypertension: a qualitative study. Nutrients.

[36] Harrison G.G., Kagawa-Singer M., Foerster S.B., Lee H., Pham Kim L., Nguyen T.U. (2005). Seizing the moment: California's opportunity to prevent nutrition-related health disparities in low-income Asian American population. Cancer.

[37] American Frozen Food Institute (2021).

[38] Yousefian Hansen A., Leighton A., Fox K., Hartley D. (2011). Understanding the rural food environment—perspectives of low-income parents. Rural Remote Health.

[39] Connors M., Bisogni C.A., Sobal J., Devine C.M. (2001). Managing values in personal food systems. Appetite.

